# Supplementary material for: Changes in clinical markers observed from pharmacist-managed cardiovascular risk reduction clinics in federally qualified health centers: A retrospective cohort study
Source: PLoS One. 2023 Mar 15;18(3):e0282940. doi: 10.1371/journal.pone.0282940 (PMC10016666; doi:10.1371/journal.pone.0282940)
Supplement: Attachment — Submitted filename: PLOS One Response to Reviewers.docx [file pone.0282940.s002.docx]

| **Comments from Reviewers** | **Author Response** |
| --- | --- |
| *Reviewer #1* | |
| In baseline characteristics the author needs to illustrate more data about other risk factors as existing cardiovascular disease, chronic kidney disease as the target control of HbA1c, systolic blood pressure, BMI and LDL targets for each | Thank you for this comment as we had also hoped to include this information. Unfortunately, these data are not collected on a routine basis and are therefore unavailable for inclusion in this report. |
| The mean follow up and p values should be enclosed in tables. | Thank you for this comment. Because of the limitations with the variability of follow-up, we only performed statistical analysis on the 3-6 month and 6-9 results. We noted the p-values with asterisks and associated comments below the figures. We also appreciate your comment about describing the follow-up. To address the concerns about the follow-up, we tried to include the number of patients contributing data to each time window for the clinical markers. While not ideal, this should give the reader a sense of the number of patients contributing data at each time point. |
| Author should include the drugs used to control each risk factor. | We agree that providing this information will help readers better understand our intervention. While these data were not collected for the purposes of the retrospective review, a statement was added to the Methods section listing commonly used medication classes. |
| Non-pharmacological methods for control risk factors as reduction of alcohol and salt consumption, body weight control and exercise recommendations. | Thank you for this suggestion. Unfortunately, these data are not consistently documented in the EHR and are unavailable for inclusion in this report. This is, however, a standard part of our care as we recommend lifestyle modifications to all patients in our care. |
| *Reviewer #2* | |
| The authors claim in the introduction section that a robust amount of evidence has established the impact of pharmacist managed CVRR clinics in reducing, and managing HbA1C, SBP/DBP and lipid levels. It is required that authors provide references for the previous studies which has provided these data for our readers. It will also be helpful if authors draw a meaningful comparison from these studies in the discussion section | Citations 10-12 support this statement; manuscript updated accordingly. |
| In the methods section, it will be helpful if authors could elaborate more on the responsibilities of pharmacists managing the CVRR clinic and services provided in these clinics. | We agree with this comment and have added a sentence to the Methods section to provide additional detail about pharmacist-managed services. |
| In the result section, for increased clarity of the readers, the authors should also provide mean values of clinical risk markers (HbA1C, SBP, DBP, LDL, non-HDL) when significant at a given point of time during the follow up, in addition to providing the mean reduction, 95% CI and p value. | Thank you for this comment, we have added the mean values to the tables under the figures and added them throughout the results section of the paper as suggested. We have also added the mean of differences and associated 95% CI for the 3-6 and 6-9 month evaluations.  We did not provide additional analysis of time points after 9 months as the declining availability of patient data and expected selection bias limits the utility of these analyses. |
| In the section “What Study Adds”, the authors should discuss the potential for reduction of major adverse cardiovascular events (MACE) with the sustained reduction in risk marker like HbA1C through the interventions of pharmacist managed CVRR clinic. | The “What Study Adds” section has been revised, as suggested to include the reduction in MACE information. |
| Lastly, in the limitation section, the authors should also acknowledge the selection bias since only patients who contributed to the follow up data by regularly following up with their appointments were included in the analysis. It is possible that these patients have healthy lifestyles, improved awareness about their disease process which might have also contributed to the reduction in their clinic markers (HbA1C). | Thank you for this suggestion. The limitations section has been revised, as suggested to include the selection bias information. |
| *Reviewer #3* | |
| 1. The author concluded that the pharmacist-managed CVRR clinic demonstrates sustained health improvements. However, because the statistical analysis is not made on data from 7 through 36 months according to Statistical Analysis in Methods section, the above-mentioned “sustained improvement” cannot be concluded or confirmed by the present study. Author should avoid and amend the conclusive description. On the other hand, I agree with possible effects on sustained health improvement instinctively. The consideration of possible sustained health improvement itself is better to be left in Discussion section. Following viewpoints are addressed in Discussion section: (1) the data presented in this study are those of patients who continue to visit the pharmacist-managed CVRR clinic with poor follow-up rate of about 70% at 3 months and 7% at 36 months; (2) drop-out patients who is potentially poor outcome is better be addressed; and (3) the continued visits to pharmacist-managed CVRR clinic may possibly be just a predictor, not an effector. | The language in the statistical analysis and methods section has been revised to clarify the shorter term sustainability of the clinical outcomes. |
| Because this study is designed as before-after study, it is basically hard to confirm that the pharmacist-managed CVRR clinic cause HbA1c improvement at 3-6 months visit. It is desired to assess possible other contributing factors such as interventions by FQHC and/or primary care provider (PCP), especially at initiation. | Information was added to the limitations section to include other contributing factors. |
| Because the statistical evaluation is only made for data at baseline and 3-6 months visit, the continuous depiction of HbA1c and other clinical markers profile from baseline through 36 months may lead readers misinterpret. Therefore, author should clearly describe that the data from 7 through 36 months are just shown as descriptive values without statistical evaluation, or it might be desirable to create a Figure dedicated to the primary and secondary outcome evaluation at 3-6 months visit separately from Figure 2-4 if possible. | Footnotes were added to each Figure to clarify the descriptive nature of data beyond six months. |
| Because pharmacist-managed CVRR clinic is not a universal system and may have diversity among county even in United States, the system and the medical care that can be provided in Indiana county are summarized in Method section. In addition, the respective role of pharmacist-managed CVRR clinic and PCP in this system are recommended to be mentioned. | A sentence was added in the Methods section to provide additional detail about pharmacist-managed services. Editor: A copy of the Collaborative Practice Agreement (31 pages) can be made available as an Appendix, if desired. Please advise. |
| Drugs and treatment that can be handled in pharmacist-managed CVRR clinic should be clearly and concretely mentioned in Method section. | A statement was added to the Methods section listing commonly used medication classes. |
| Since there seems to be a difference between the description in Statistical Analysis and the description in Result section and Figures, it is desirable to attach a protocol which is approved by the Ethics Committee and/or statistical analysis plan as Supplement. | We have provided additional detail throughout the statistical analysis section to better explain the results which are described. All of the proposed evaluations fall within the approved retrospective, de-identified data evaluation reviewed by the IRB. |
| Because reference 14 has no description of sample size calculation, author probably cite reference 14 to obtain the mean and standard deviation of HbA1c before and after treatment.　Author should demonstrate concrete HbA1c values for sample size calculation. In addition, because “reduction of 0.5 points” would be “reduction of 0.5 percent”, and no description of page number of reference 14 in Reference section, author should correct them appropriately. | Thank you for this comment. Yes, we used reference 14 to obtain the mean and SD for our sample size calculation. We have added the page number to the reference and modified the text accordingly.  We have also added this reference:  de Barra M, Scott CL, Scott NW, Johnston M, de Bruin M, Nkansah N, Bond CM, Matheson CI, Rackow P,Williams AJ, Watson MC.  Pharmacist services for non-hospitalised patients.  Cochrane Database of Systematic Reviews 2018, Issue 9. Art. No.: CD013102. |
| Because statistical analysis is not conducted to data at from 7 through 36 months visits, the description of analysis of data at 6-9 months visit should be avoided in Result section and Figures. | Footnotes were added to each Figure to clarify the descriptive nature of data beyond nine months. Although, the data beyond nine months is descriptive in nature, authors feel that inclusion is still warranted. We also noted that the evaluation of 6-9 month outcomes was a secondary outcome that we planned on analyzing within the methods section. |
| Author should clarify the handling of data at months in multiples of 3. For instance, if data at 6 months visit includes to 3-6 months period, description of 6-9 months should be corrected to 7-9 months. Similar correction should be made throughout the manuscript and Figures. | We applied a standard procedure to all the data to calculate which interval the result was closer to when deciding which interval to attribute the result to. The methods section has been updated to clarify, as suggested. |
| Author should demonstrate the rule of handling data in the following cases in Statistical Analysis in Methods section: (1) patient visits pharmacist-managed CVRR clinic more than twice during each 3 months period; and (2) data at 4 and at 5 months visits. | The number of visits was not collected, but rather the clinical markers within the specified time frames. Please see the comment above for more detailed answers to your insightful comment. |
| Author should amend followings in Figure 1: (1) because “Consort diagram” and “Allocation” are basically for terms of randomized clinical trial, it is better to be amended appropriately; and (2) because the fact that 631 patients are registered and 445 patients are able to evaluate at 3-6 months visit means that 186 patients dropped out from the study, the detail of drop out should be described. | Thank you for your comment. We have amended the CONSORT diagram, as suggested.  To clarify issues regarding point 2 about 186 patients dropping out, one of the criteria for enrollment was that they had to provide at least one point of follow-up data after three months to be eligible for this analysis. During our analysis we made sure that all patients had additional follow-up beyond the baseline encounter. It is for that reason that the 186 patients did not actually drop out, but did not have an HbA1c measurement within the 3-6 month time window. All of these patients had a later HbA1c measurement after the 3-6 month window which is why they were still included as eligible within this analysis.  Regarding “dropout”-We have added a comment to the limitations section as we are not able to precisely determine who has dropped out of the clinic as many patients “graduate” out of the more intense clinical pharmacist support and return to routine clinical care. Unfortunately, these dynamics make it difficult to determine which patients are truly lost to follow up as that information was not routinely tracked by within the electronic medical record used for this assessment.  The limitations section has been revised accordingly, as suggested. |
| The doubled figure of Non-HDL Cholesterol should be omitted in Figure 4. | The doubled Non-HDL figure has been deleted, as suggested. |
| Reference 7 is now searched usually. Correct description. | Reference 7 has been revised, as suggested. |
| *Journal Requirements* | |
| Please ensure that your manuscript meets PLOS ONE's style requirements, including those for file naming. The PLOS ONE style templates can be found at <https://journals.plos.org/plosone/s/file?id=wjVg/PLOSOne_formatting_sample_main_body.pdf> and [https://journals.plos.org/plosone/s/file?id=ba62/PLOSOne_formatting_sample_title_authors_affiliations.pdf](about:blank) | Manuscript has been revised to meet PLOS ONE style requirements |
| In ethics statement in the manuscript and in the online submission form, please provide additional information about the patient records/samples used in your retrospective study. Specifically, please ensure that you have discussed whether all data/samples were fully anonymized before you accessed them and/or whether the IRB or ethics committee waived the requirement for informed consent. If patients provided informed written consent to have data/samples from their medical records used in research, please include this information. | This analysis extracted de-identified data from the electronic medical record these clinics use to support care. All records used in this study were de-identified prior to being included as a research record.  Because of our approach, the IRB protocol was approved as exempt and waived the requirements as informed consent. |
| We note that you have indicated that data from this study are available upon request. PLOS only allows data to be available upon request if there are legal or ethical restrictions on sharing data publicly. For information on unacceptable data access restrictions, please see <http://journals.plos.org/plosone/s/data-availability#loc-unacceptable-data-access-restrictions>. | The authors need special permission to share the data set. This is not one of the provisions within the IRB protocol approval.  Additional permissions from the IRB and the health system would be required to submit the data to a public repository.  Authors are willing to revise and resubmit the IRB application and request health system approval if required for the manuscript to be published, but did not want to delay the initial resubmission process here. At a minimum, the de-identified data set can be shared with any investigators who make reasonable requests for this information. |
| In your revised cover letter, please address the following prompts: If there are ethical or legal restrictions on sharing a de-identified data set, please explain them in detail (e.g., data contain potentially identifying or sensitive patient information) and who has imposed them (e.g., an ethics committee). Please also provide contact information for a data access committee, ethics committee, or other institutional body to which data requests may be sent. | The cover letter includes the requested information. |
