# Supplementary material for: Changes in clinical markers observed from pharmacist-managed cardiovascular risk reduction clinics in federally qualified health centers: A retrospective cohort study
Source: PLoS One. 2023 Mar 15;18(3):e0282940. doi: 10.1371/journal.pone.0282940 (PMC10016666; doi:10.1371/journal.pone.0282940)
Supplement: Attachment — Submitted filename: Response to Reviewers 4.5.22.docx [file pone.0282940.s003.docx]

| **Reviewer Comments** | **Response from Authors** |
| --- | --- |
| Have the authors made all data underlying the findings in their manuscript fully available? The data should be provided as part of the manuscript or its supporting information, or deposited to a public repository. | The deidentified data set has been provided. Please advise if additional information is necessary. |
| **Reviewer 1** | |
| Risk factors as presence of cardiovascular disease, cerebrovascular disease or peripheral vascular disease should be included in the baseline characteristics | Authors agree that these data points would have been a strong addition to the patient characteristics. Unfortunately, these data were not collected and cannot be incorporated. |
| fasting and random blood sugar measurement should be mentioned as a standard of blood sugar control | Fasting and random BG data were not collected as there is inconsistency in and lack of documentation of this information.  This statement was added to the Limitations section to address this comment: “While fasting and random blood glucose values would have added beneficial insight into overall diabetes management, including the incidence of hypoglycemia, these data were not included in this analysis due to the inconsistency in documentation and reporting of this information in the electronic medical record.” |
| presence of hypoglycemia not mentioned | Incidence of hypoglycemia was not collected as there is inconsistency in and lack of documentation of this information.  This statement was added to the Limitations section to address this comment: “While fasting and random blood glucose values would have added beneficial insight into overall diabetes management, including the incidence of hypoglycemia, these data were not included in this analysis due to the inconsistency in documentation and reporting of this information in the electronic medical record.” |
| the types of medication insulin, oral hypoglycemic or dietary control are not clear. | This statement was added to the Limitations section to address this comment: “Furthermore, specific medication changes and dietary patterns were beyond the scope of this analysis and therefore not included. However, the pharmacists managing medications in this study follow evidence-based recommendations, prioritize first-line medications where appropriate, and limit barriers such as clinical inertia.”  This reference was also added to support the above statement: Meredith AH, Buatois E, Krenz J, et al. Assessment of clinical inertia in people with diabetes within primary care. J Eval Clin Pract. 2021 Apr;27(2):365-370. |
| many underlying conditioned as anemia and hyperthyroidism could affect the accuracy of HBA1C thus, it should be excluded if present. | This statement was added to the Limitations section to address this comment: “HbA1C was identified as the best, standardized measure to report for the purposes of this study. However, underlying conditions that could potentially affect the accuracy of HbA1C values, such as anemia or thyroid conditions, were not collected.” |
| **Reviewer 3** | |
| The author may mention the possibility that the patient insurance status might affect the results especially in underinsured patients. Since uninsured patients may tend to avoid expensive medical care, it might be pronounced the importance of being able to improve and maintain 2% reduction in HbA1c even the value itself is considered as poor HbA1c control situation. | Authors have included this statement, which addresses this comment: “Given that the majority of patients enrolled in the CVRR service within our health-system are uninsured or have public insurance, any savings related to the care of these patients are also savings to the state and federal governments and taxpayers. Even accounting for the cost of providing the service, clinical pharmacists have demonstrated a positive return-on-investment [1].” |
| The follow-up rate at 36 months should be mentioned in Result section | The following statement was added to the Results section to address this comment: At 36 months, 9.7% (N=43) of the initial study population were eligible for evaluation. |
| Regarding Table 1, the author should clearly state more detailed rule of classifying blood pressure in the case that the systolic and diastolic blood pressure belong to different subgroups such as 130/70 mmHg. The author should state the rule to treat blood pressure in Statistical Analysis section. | The following statement was added to the Statistical Analysis section to address this comment: “BP was classified by the highest SBP or DBP reading into the following categories: <120/80, 120-139/80-89, 140-159/90-99, and 160-179/100-110, and >180/110.” |
| As the caption of Fig.1, “Consort Diagram. Patient characteristics” is inappropriate. The author should consider to change to “Study diagram” | Revised, as suggested. |
| “Secondary outcomes” in line 194 should be amended as subheading using Bold font and appropriate letter size. | Revised, as suggested. |
| “A1C” in line 243 should be corrected to “HbA1C”. | Revised, as suggested. |
| According to the response to reviewers and revised manuscript, the statistical analysis is not conducted to data at from 9 through 36 months visits. The description of footnote in Figure 2 and 3 should be corrected so that it does not contradict. | Changed footnotes on Fig 2 and 3 to say 9 months instead of 7, as suggested. |
| According to the description that the additional statistical analyses were not performed for the outcome measurements captured between 9 months and 36 months, the time window of secondary endpoint should be 6-8 months and the time window of primary endpoint should be 3-5 months. The author should check again and correct description if necessary. | Time frames have been adjusted throughout the manuscript and in the figures, as suggested. |
| The author stated “The external validity of this study is strengthened by the multi-site nature of the data” in line 236. However, because no external validation was conducted in this study, this sentence should be omitted. | This sentence has been deleted, as suggested. |
