# Supplementary material for: Changes in clinical markers observed from pharmacist-managed cardiovascular risk reduction clinics in federally qualified health centers: A retrospective cohort study
Source: PLoS One. 2023 Mar 15;18(3):e0282940. doi: 10.1371/journal.pone.0282940 (PMC10016666; doi:10.1371/journal.pone.0282940)
Supplement: Attachment — Submitted filename: Response to Reviewers.docx [file pone.0282940.s004.docx]

| **Reviewer Comment** | **How Addressed** |
| --- | --- |
| Introduction: It would be of interest to reinforce the need of more evidence that justifies the study. The sentence “A robust amount of evidence has established the impact of pharmacists in reducing and managing HbA1C levels, systolic and diastolic blood pressure, and lipid levels to mitigate cardiovascular risks [10-12]” is wrongly pointing in the opposite direction as you say there is a lot of evidence on several topics, including diabetes (which would not justify your study), which is not totally correct and is not relating to the references used (that show only some evidence on DM). This should be rewritten to express the idea that there is some evidence of interest showing that pharmacists’ interventions may result in improvements of several cardiovascular variables but further research is needed specifically in the field of DM management, and that it would be of interest or specially needed for specific population subgroups at risk (e.g. the population in your study). As for the next phrase “However…” please remove the word “however”. | Paragraph 2 of the introduction section has been modified to read:  “Some evidence has established the impact of pharmacists in improving several cardiovascular clinical markers, such as reductions in HbA1C levels, systolic and diastolic blood pressure, and lipid levels [10-12]. These studies typically encompass six months to two years and do not focus on populations who are underserved. The result is a lack of literature surrounding the long-term cardiovascular and cost-effective benefits of pharmacist-management of cardiovascular-related diseases in populations who are under resourced.” |
| As for the “Statistical analysis” subsection, it starts enumerating and defining the variables collected and related issues (which is not statistical analysis). Thus, it would improve if this title is changed for “Variables and statistical analysis” | Section title changed as suggested. |
| In the “statistical analysis” subsection, there is a minor error that should be changed: “140-159/90-99, and 160-179/100-110, and >180/110.” --> “140-159/90-99, 160-179/100-110, and >180/110.” | Values have been changed to:  <120/80, 120-139/80-89, 140-159/90-99, and 160-179/100-109, and ≥180/110 |
| Results section. Table 1… in the first cell of the second column you write “n (%)” but below you also show variables with mean and (SD). Thus, you should change it for something generic such as “Total sample (n=your sample size)” and for each variable include (n, %). E.g.: "Gender (n, %)". Also, for the variables where you indicate (mean, SD) and (n,%), you should name the variable in a manner that the reader can easily identify what your are showing… E.g.: one row with “Age (mean, SD)” and another row with “Age (n, %). Please also change “characteristic” to a more appropriate wording. | The overall results column has been changed to a heading of “Results (n=631)”. Subsequent cells/rows have been modified to include the “n (%)”, as appropriate. |
| 1. Methods section of the abstract Please change “All patients seen by a CVRR pharmacist over a 5-year period were evaluated” to something similar to the information you provide in the article… e.g.: “Data were collected from patients seen by a CVRR pharmacist within the timeframe of January 1, 2015 through February 28, 2020” (or something similar). ALSO, add something regarding “timeline” of the analysis e.g. “HbA1C from baseline after 3 to 5 months was assessed with pared t-tests analysis. Other clinical variables were assessed and additional analysis were performed at 6-8 months. Additional results are reported between 9 months and 36 months of follow up”. Something in this line would help the reader to understand what you did. | The methods section of the abstract has been updated to read:  “This retrospective cohort chart review occurred in eight pharmacist-managed CVRR federally qualified health clinics (FQHC) in Indiana, United States. Data were collected from patients seen by a CVRR pharmacist within the timeframe of January 1, 2015 through February 28, 2020. Data collected include: demographic characteristics and clinical markers between baseline and follow-up. HbA1C from baseline after 3 to 5 months was assessed with pared t-tests analysis. Other clinical variables were assessed and additional analysis were performed at 6-8 months. Additional results are reported between 9 months and 36 months of follow up.” |
| 2. Dipeptidyl peptidase 4 inhibitors Please state in the introduction section that dipeptidyl peptidase 4 inhibitors were used (if they were) or alternatively mention this as a limitation in the discussion as the restriction of therapy available to patients may impact on the results compared with other practice. | DPP4s have been added to the list of commonly prescribed medications in the introduction section. |
| The reviewer states the importance of follow-up problems. Please state a sentence in the results sections of the ABSTRACT with this regard. E.g. The follow-up losses were XX% at 3-5 months and YY at ZZ months. Also, in the conclusion section of the abstract you should state something such as “the great proportion of loss to follow-up is a limitation of this study to be considered”. Also, as recommended by one of the reviewers, please explain these problems and their implications in the discussion section as a limitation. | The abstract results have been modified to include the statement:  “The follow-up losses were 29.5% at 3-5 months and 93.2% at 33-36 months.”  The abstract conclusion has been modified to include the suggested statement, and further described in the limitation section of the discussion. |
| Reviewer #1: i accept the manuscript PONE-D-21-08257R3 titled " Changes in Clinical Markers Observed from Pharmacist-Managed Cardiovascular Risk Reduction Clinics in Federally Qualified Health Centers: A Retrospective Cohort Study" for publication. | Noted. Thank you. |
| Reviewer #3: The author intends to demonstrate the benefit of pharmacist-managed cardiovascular risk reduction (CVRR) clinics. Although the collected data itself is surely valuable, the adopted population is unique because of non-negligible poor follow-up rate (70% at 3-5 months; 50% at 6-9 months; and 30% at 12-15 months). The author should mention that the findings in the present study was the observation in such relatively minor population who continuously visit CVRR clinic, otherwise the reader can be misinterpreted. | The “Main Findings of the Study” has been modified so that the first sentence reads:  “The results of this study confirm that patients who consistently received care from pharmacist-managed CVRR services in FQHCs demonstrate…”.  The “What the Study Adds” has been modified to include the statement:  “…there is strong potential for the reduction of major adverse cardiovascular events (MACE) for the high-risk patients seen in these clinics who maintain follow-up over time.”  The limitations section also discusses selection bias, highlighting that the results apply to those that continue to receive care. |
| In Abstract, the author described “All patients seen by a CVRR pharmacist over a 5-year period were evaluated” without any description regarding statistically analysis, while the author described only regarding the findings at 3-5 months and at 6-8 months. This inconsistent and insufficient description should be amended. | Addressed in comment above |
| In Introduction section: Are dipeptidyl peptidase 4 inhibitors not allowed in CVRR clinic? | Addressed in comment above |
| In Discussion section, Main findings of the study paragraph: Regarding the description “The results … six months”, the author should mention that this finding was observed in about half of the populations who continuously attended CVRR clinic. | Addressed in comment above |
| In Discussion section, What the study add paragraph: Regarding the description “Given the reduction … these clinics.”, the author should also mention that this potential was for about half of the populations who continuously attended CVRR clinic. | Addressed in comment above |
| As the reviewer mentioned continuously, the small follow-up rate at CVRR clinic is non-negligible issue. The followings should be addressed and mentioned: (1) the author’s opinion regarding the benefit of education in CVRR clinic for the discharged patients who achieved their clinical goals; and (2) assessment of poor follow-up rate in Discussion section (the reviewer recommends to move relevant issue from Limitation section) | (1) A statement has been added to the “What the study adds” section to read:  “Additionally, the hope is that patients who are discharged from CVRR services due to achievement of clinical goals are able to sustain healthy outcomes due to the knowledge gained during their interactions with the CVRR pharmacist.”  (2) Thank you for the comment. The authors feel that it is most appropriate to leave in the limitations section at this time. |
| Well done on this important research study with insightful findings. You have clearly acknowledged all the limitations with your paper which should prompt further research. | Noted. Thank you. |
